# Network Evolution Induced by Asynchronous Stimuli through Spike-Timing-Dependent Plasticity

**DOI:** 10.1371/journal.pone.0084644

**Published:** 2013-12-31

**Authors:** Wu-Jie Yuan, Jian-Fang Zhou, Changsong Zhou

**Affiliations:** 1 College of Physics and Electronic Information, Huaibei Normal University, Huaibei, China; 2 Department of Physics, Centre for Nonlinear Studies and the Beijing-Hong Kong-Singapore Joint Centre for Nonlinear and Complex Systems (Hong Kong), Institute of Computational and Theoretical Studies, Hong Kong Baptist University, Kowloon Tong, Hong Kong; 3 Beijing Computational Science Research Center, Beijing, China; 4 Research Centre, HKBU Institute of Research and Continuing Education, Virtual University Park Building, South Area Hi-tech Industrial Park, Shenzhen, China; University of Maribor, Slovenia

## Abstract

In sensory neural system, external asynchronous stimuli play an important role in perceptual learning, associative memory and map development. However, the organization of structure and dynamics of neural networks induced by external asynchronous stimuli are not well understood. Spike-timing-dependent plasticity (STDP) is a typical synaptic plasticity that has been extensively found in the sensory systems and that has received much theoretical attention. This synaptic plasticity is highly sensitive to correlations between pre- and postsynaptic firings. Thus, STDP is expected to play an important role in response to external asynchronous stimuli, which can induce segregative pre- and postsynaptic firings. In this paper, we study the impact of external asynchronous stimuli on the organization of structure and dynamics of neural networks through STDP. We construct a two-dimensional spatial neural network model with local connectivity and sparseness, and use external currents to stimulate alternately on different spatial layers. The adopted external currents imposed alternately on spatial layers can be here regarded as external asynchronous stimuli. Through extensive numerical simulations, we focus on the effects of stimulus number and inter-stimulus timing on synaptic connecting weights and the property of propagation dynamics in the resulting network structure. Interestingly, the resulting feedforward structure induced by stimulus-dependent asynchronous firings and its propagation dynamics reflect both the underlying property of STDP. The results imply a possible important role of STDP in generating feedforward structure and collective propagation activity required for experience-dependent map plasticity in developing in vivo sensory pathways and cortices. The relevance of the results to cue-triggered recall of learned temporal sequences, an important cognitive function, is briefly discussed as well. Furthermore, this finding suggests a potential application for examining STDP by measuring neural population activity in a cultured neural network.

## Introduction

In neural systems, an asynchronous state characterized by arbitrarily low mean spiking correlations has been observed extensively in experiments [1], [2], which offers substantial advantages for information processing and coding [2]. Especially in sensory systems, the asynchronous firings or external asynchronous stimuli (which can produce the asynchronous firings) play an important role in perceptual learning, associative memory and map development [3], [4]. It has been experimentally found that, the sensory asynchronous stimuli are used to mediate the plasticity of neural responses for learning and memory in adults and the activity-dependent development of sensory map during a critical period of early postnatal life [3]–[8]. For instance, late visual stimulus is able to serve instructive role for earlier arriving auditory input in the barn owl, a highly efficient predator [5]. Neural associations between stimuli and reward expectancy in primary visual cortex can form when adult rats experience an association between visual activity (conditioned stimulus) and subsequent reward (unconditioned stimulus) [6]. A recent study has reported that the asynchronous stimuli producing binocular retinal activities in mice during a critical period in development enhance eye-specific segregation and regulate retinotopy in the developing visual system [7].

Recently, an important cognitive function, cue-triggered recall of learned temporal sequences, has been experimentally studied [9], [10]. The cue-triggered recall of a learned temporal sequential firing has been found in neuronal ensemble of hippocampus, its surrounding cortical areas [11], [12] and visual cortical circuits [13]. Importantly, the learned sequential firing can be trained in experiments by the repeated asynchronous stimuli. For example, a recent study reported that [13], a moving spot that asynchronously stimulated the neurons whose receptive fields fall along the motion path, can evoke the sequential firing of neuronal ensemble in primary visual cortex. Shortly after repeated motion conditioning, a brief flash at the starting point of the motion path can also evoke the sequential firing similar to that evoked by the moving spot. In this experiment, the sequence learning and cue-triggered recall become more persistent with more repeats of the stimulation of the moving spot. This indicates that, asynchronous stimuli evoked by the moving spot, play an important role in the formation of the sequence learning and recall.

In general, asynchronous stimuli can be used to mediate the activity-dependent map development during a critical period of early postnatal life and the synaptic plasticity for learning and memory in adults [3], [4], [6], [8], [14]. Especially, asynchronous stimuli contribute to the formation of sequence learning and recall [11]–[13]. Many neuron scientists thought that, these functional roles result from activity-dependent synaptic plasticity between neurons [4], [5], [7], [8], [13]. However, the organization of structure and dynamics of neural networks due to synaptic plasticity are not well understood in the presence of asynchronous stimuli. It is clear that, the asynchronous stimuli can produce asynchronous, segregative firings of pre- and postsynaptic neurons. So far, experiments have extensively found a type of synaptic modification: spike-timing-dependent plasticity (STDP), which is highly sensitive to correlations between pre- and postsynaptic firings [15]–[17]. Particularly, this synaptic plasticity has been widely found in sensory systems [18]–[21], which could contribute to learning, memory and development [4], [21]–[23]. Therefore, it is natural to expect that STDP could play an important role in response to asynchronous stimuli.

In order to explore the role of STDP in the presence of external asynchronous stimuli, we construct a network model with local connectivity and sparseness depending on the spatial distance between neurons, and use external currents to stimulate alternately on different spatial layers. We here expect that, STDP can modify some synaptic strengths to form a feedforward network due to asymmetric pre-post spike orderings resulting from asynchronous stimulus pairings. In the resulting feedforward structure, a short stimulation current can be expected to produce wave propagation of neuronal firings, which could provide an alternative explanation for the cue-triggered recall of sequence learning. In addition, the results imply an important role of STDP in generating feedforward structure and collective propagation activity required for experience-dependent map plasticity in developing in vivo sensory pathways and cortices. Since the synaptic modification due to STDP is sensitive to correlations between pre- and postsynaptic firings [15], [16], the resulting feedforward structure is expected to reflect the property of STDP. In the feedforward network, directed propagation dynamics of neural activity is also expected to reflect the underlying property of STDP, which suggests an application of the analysis for examining STDP by measuring neural population activity in cultured neural network.

## Materials and Methods

### Spatial Neural Network Model

**Figure 1:**
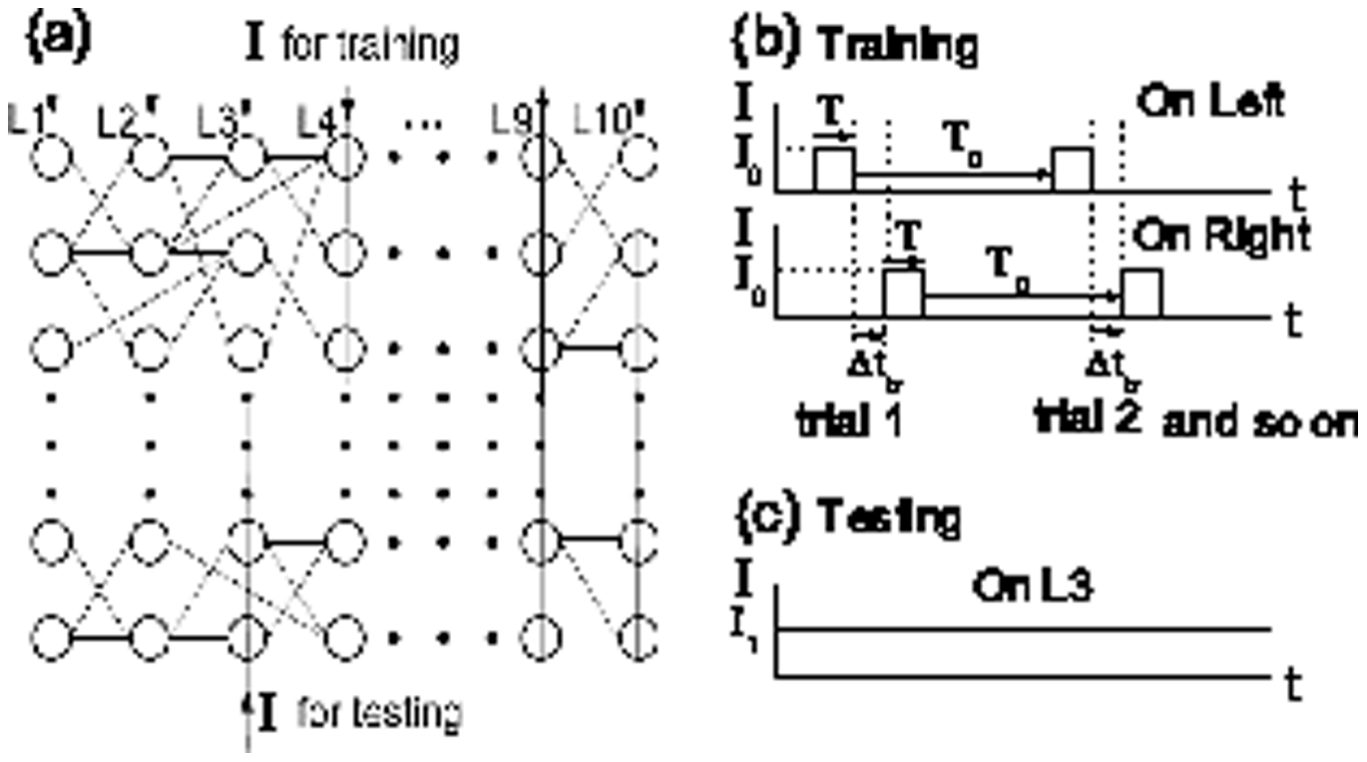
The locally-linked neuronal network model and external asynchronous stimulus currents. (a) The locally-linked neuronal network model on two-dimensional square having 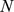 neurons and 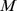 layers. Here, we take 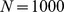, 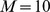 and label neural number (1 – 1000) from left to right layers. (b) To train the network, the input pulse current 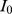 with duration 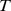 is injected alternately into each pair of layers with the left-right sequence having the same inter-stimulus interval 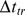, respectively. After each training trial, there is a long enough time 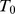 to let network activity recover to the rest state for the next training. We perform training for all the pair of layers with the same number of trials. (c) To test the resulting feedforward structure and its propagative capacity, a steady current 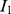 is injected into a certain layer (here, we choose the 3rd layer, i.e., L3).

Recently, spatial network has been widely modeled [24]–[27]. Considering the locally-linked structure in real neural systems, we here construct a two-dimensional neural network model with local connectivity and sparseness, depending on the spatial distance between connected neurons. As shown in Fig. 1(a), 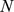 neurons are located in lattice points of the two-dimensional square with 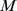 layers, where each layer has 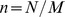 neurons. Each pair of neurons is connected locally with probability 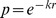 [28] by synapse with random-chosen initial synaptic strength from 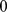 to 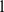, where 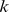 denotes the locally connecting strength and 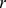 is the distance between two neurons (the side length of square is regarded as 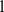 here). For simplicity but without loss of generality, the model is composed of Integrate-and-Fire type neurons with chemical couplings of 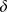 function. The dynamics of the membrane potential 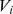 of neuron 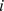 is described by
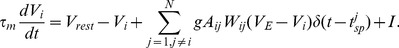
(1)


Here, we adopted the parameter values as those in Refs. [29], [30], which model V1 cells according to empirical observations [31]. The membrane time constant 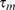 equals 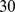 ms, the resting potential 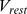 is 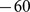 mV, and the reversal potentials 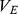 for all the excitatory synapses are 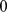 mV. Each neuron 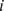 integrates external stimulus 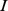 and inputs coming from the connecting neurons 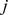 at spike time 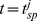. When the potential 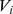 reaches the threshold value 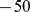 mV, the neuron 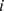 emits a spike, and then the membrane potential is reset to the resting potential 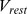. Here, we do not consider the refractory period of neuronal activity in order to improve the speed of modeling. However, the qualitative results are independent of the refractory period. Also, the parameter 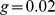 is constant synaptic conductance, 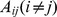 is the adjacency matrix (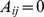 or 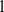) and 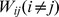 is the weight of synaptic strength from neurons 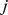 to 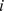. The synaptic modifications are subject to STDP, which will be described in the following.

### Spike-Timing-Dependent Plasticity

STDP has been extensively found in experiments and has received much theoretical attention in recent years [32]–[36]. The temporal order of presynaptic and postsynaptic spikes determines whether the synaptic change is potentiated or depressed. The synaptic change will be potentiated if presynaptic neuron spikes before postsynaptic neuron; otherwise, it will be depressed. The changes of synaptic strength are a function of the relative timing between presynaptic spike arrival and postsynaptic firing. The smaller the lag between the spikes, the larger the change. The modification 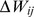 of synaptic weight 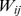 from neuron 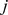 to 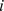 is approximated by exponential functions of the spiking time difference 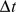 between post- and presynaptic neurons [15],
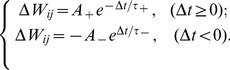
(2)


The parameters 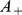, 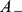, 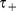 and 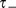 describe the property of STDP. Generally, STDP can be divided into two forms in numerical studies. If 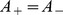 and 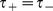, it is temporally symmetric form; otherwise, it is temporally asymmetric form. According to Eq. (2), the synaptic dynamics in Eq. (1) is described as,
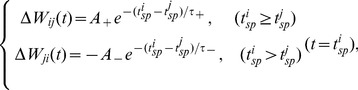
(3)when neuron 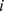 fires. For simplicity of simulation and without loss of generality, we only consider the low-frequency stimuli. Thus, we here consider only the largest modification corresponding to last spike time 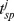 of neuron 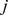 at spiking time 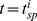 of neuron 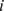 (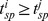). Particularly, at 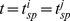 (i.e., synchronous spikes of neurons 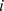 and 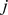), 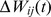 and 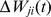 are both equal to 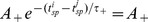. As discussed in [15], 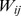 (or 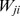) is set to 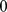 if the change makes 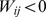 (or 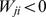) and 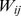 (or 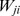) is set to the maximal value 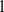 if the change would make 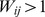 (or 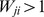).

For STDP model, the authors only consider.

### External Asynchronous Stimuli

For many years, the neural-response to external stimulus has been challenging hot topics for the study of neurology [5], [7], [37], [38]. By using external asynchronous stimuli, our model produces a feedforward network due to STDP. In our model, for each pair of layers, we firstly stimulate the left layer with duration 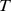 and then stimulate the corresponding right layer with the same duration 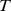 after time 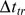 by inputting pulse current 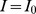 (Fig. 1(b)), where 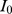 is set as a small enough value that it cannot induce spikes of neurons in other layers. Clearly, the synapses from neurons of the left layer to those of the right layer are potentiated by STDP because spikes of the left neurons precede those of the right neurons due to interval 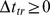, while the synaptic strengths of reverse connections are depressed. After each training trial for each pair of layers, there are long enough time 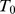 to let network activity recover to the rest state for the next training. Here, we do the training with the same number of trials for each pair of layers. After many training trials for all the pairs of layers, all the synaptic weights from left layers to right layers will increase almost to 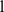, while reverse synaptic weights will decrease approximately to 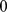. Namely, the resulting network structure is expected to be a feedforward structure from left to right after many enough training trials. Then, to test the feedforward structure, we measure collective propagation property of neural activity by injecting a steady current 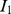 into a certain layer in the resulting network (Fig. 1(c); here, we choose the 3rd layer).

## Results

### Generation of Feedforward Structure

**Figure 2:**
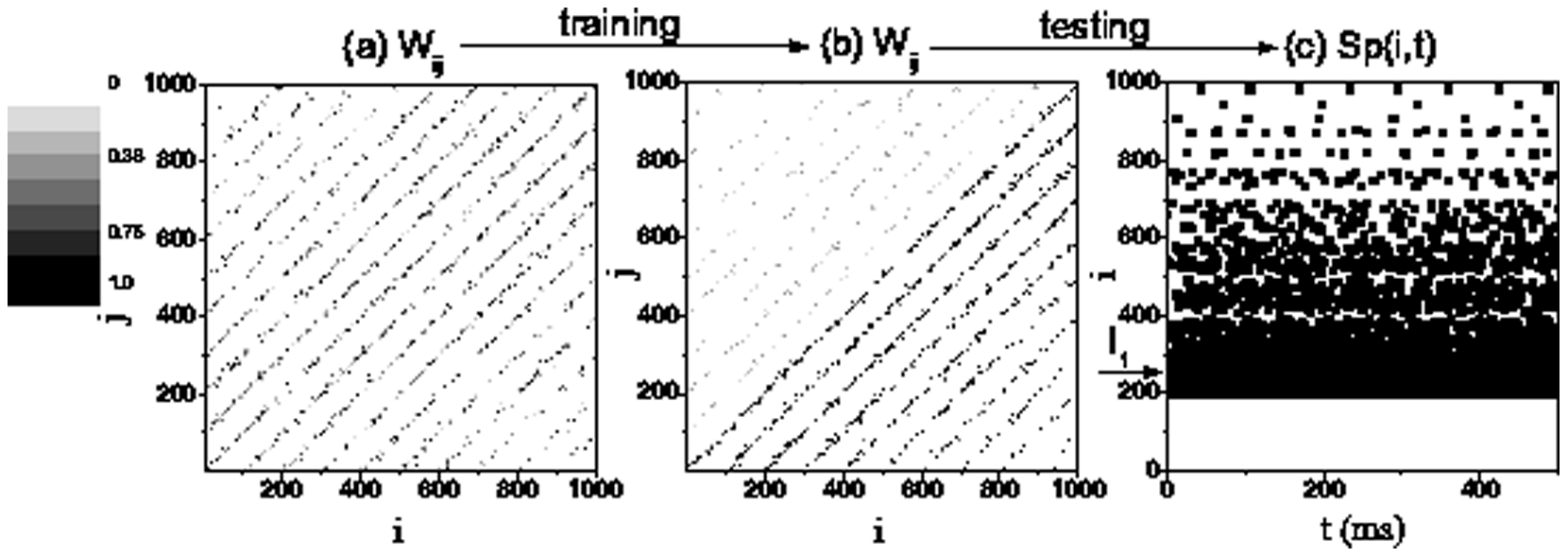
Evolution of the network and dynamical propagation. The weights of synaptic strengths on the model before (a) and after (b) 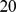 training trials. (c) The spatiotemporal pattern 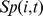 of neuron spikes by injecting steady current for testing feedforward network. The system parameters are 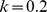, 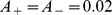 and 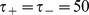 ms. The parameters for training (Fig. 1(b)) and testing (Fig. 1(c)) are given by 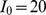, 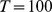 ms, 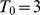 s, 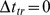 ms, and 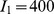.

We firstly simulate the generation of feedforward structure. As shown in Fig. 2, the initially locally-linked network (Fig. 2(a)) forms a feedforward structure (Fig. 2(b)) due to STDP by the training procedure mentioned above. Moreover, we show in Fig. 2(c) the propagation activity of neural spikes by inputting steady current. Obviously, the signals from the stimulated layer 3 (neurons 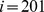 to 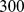) spread only to the right layers (neurons 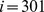 to 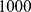), while the left layers (neurons 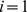 to 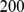) cannot respond. This directed propagative activity indicates the formation of feedforward structure and thus reflects the existing of STDP in this neural network.

### Effect of Number of Training Trials

**Figure 3:**
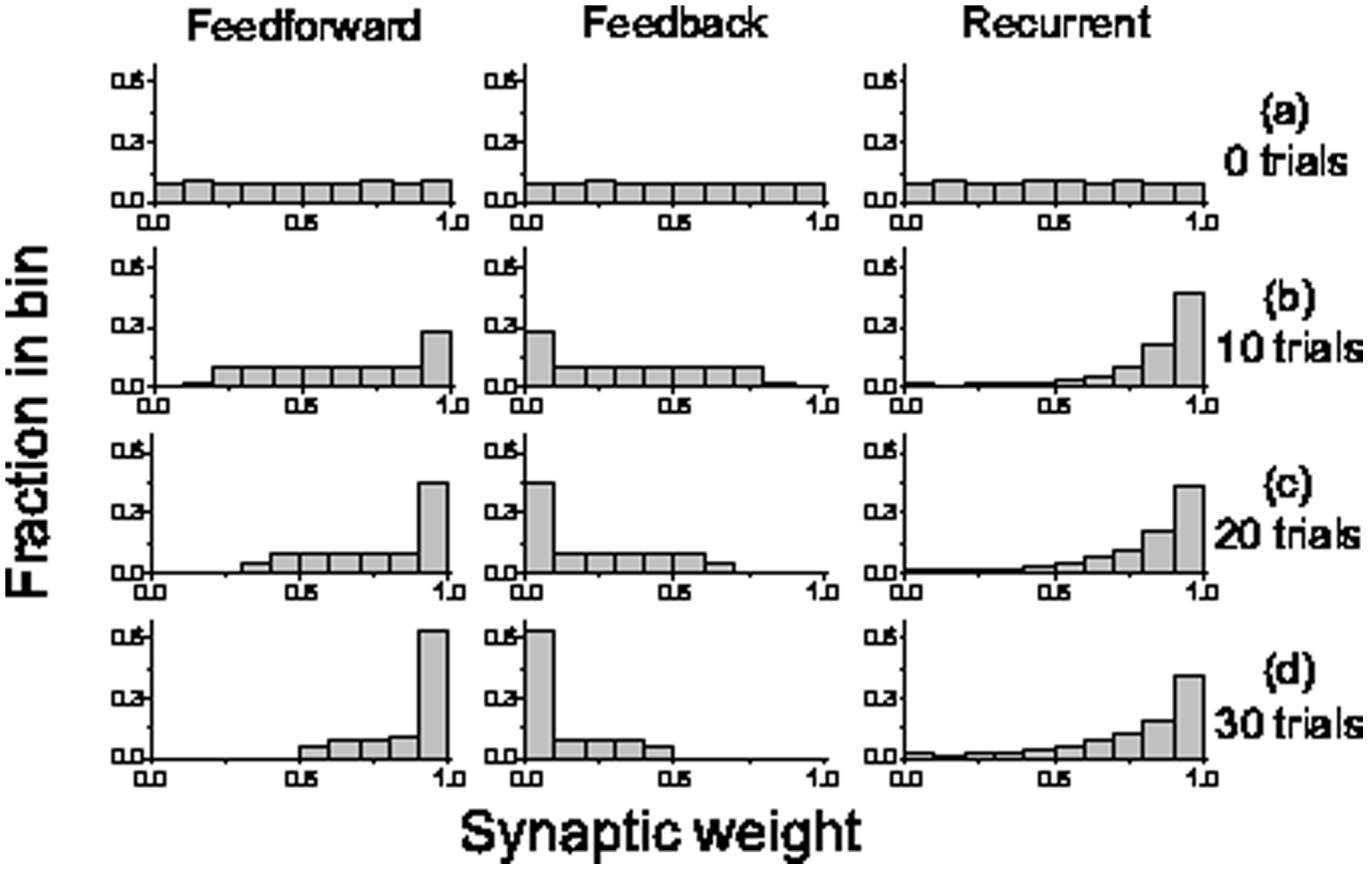
Distribution of synaptic weights after the different training trials. Distribution of synaptic weights for three different connection types: feedforward (left), feedback (middle) and recurrent (right) at the beginning of training (a) and after the training with different number of trials 10 (b), 20 (c), 30 (d). The same parameters are the same as in Fig. 2, except for 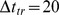 ms.

In order to further explore the impact of the training on the resulting feedforward structure, we next investigate the dependance of synaptic weights for different connection types on the number of training trials while keeping 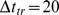 ms. Here, we divide the synaptic connections into three types: feedforward (in the training direction, from left layer to right layer in model Fig. 1(a)), feedback (the opposite training direction, from right layer to left layer) and recurrent (intra-layer) connections. Fig. 3(a) shows the situation at the beginning of our simulations, with all synaptic weights randomly chosen from 0 to 1. After 10 trials (Fig. 3(b)), the feedforward synaptic weights are potentiated by STDP because left (presynaptic) neuron spikes before right (postsynaptic) neurons. On the contrary, the feedback synaptic weights are depressed due to reverse pre-post spike ordering. In addition, we can see the strong increase of recurrent synaptic weights. This is because the linked neurons within a layer often synchronously fire due to the same driving stimulus, causing the synaptic potentiation by STDP (see Eq. 3 for 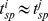). After 20 (Fig. 3(c))and 30 (Fig. 3(d)) trials, many more feedforward synapses have been strengthened (e.g. 63% being approximate to 1 in the left panel of Fig. 3(d)) and many feedback synapses have been weakened (e.g. 63% being approximate to 0 in the middle panel of Fig. 3(d)), which makes the network form feedforward structure (Fig. 2(b)).

**Figure 4:**
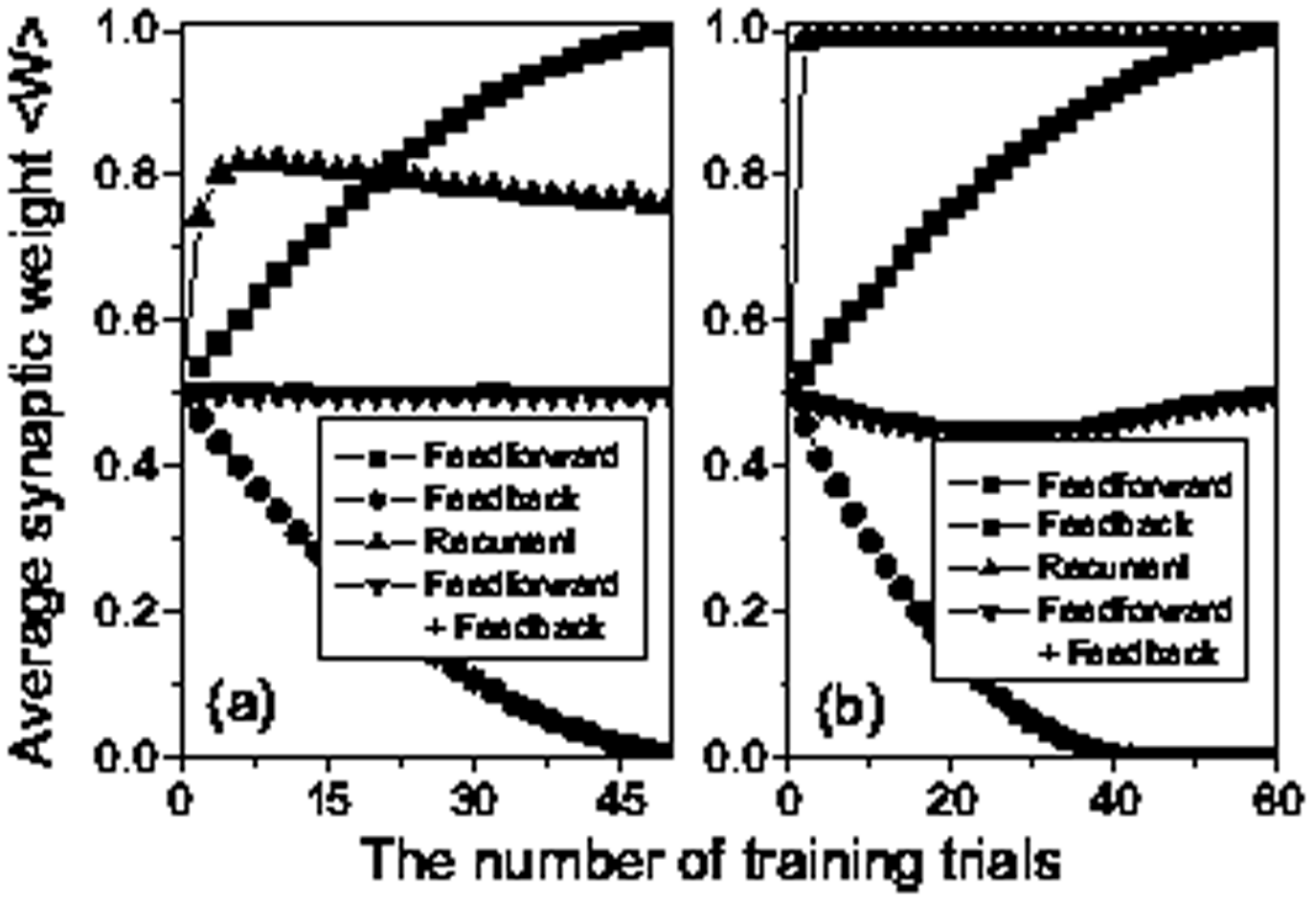
Average synaptic weights as a function of the number of training trials. Average synaptic weights for three different connection types: feedforward (squares), feedback (circles) and recurrent (upward triangles) as a function of the trial number for temporally symmetric form of STDP (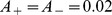 and 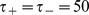 ms) (a) and for temporally asymmetric form of STDP (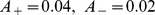 and 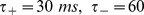 ms) (b). The other parameters are the same as in Fig. 2 except for 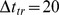 ms. Also, we show the average synaptic weights for the connections including both feedforward and feedback types (downward triangles) in (a) and (b).

Fig. 4(a) shows clearly the changes of average synaptic weights with respect to the number of training trials. Obviously, the increasing amount of synaptic modification for feedforward connections is equal to the decreasing amount of synaptic modification for feedback connections (Fig. 4(a)), which results from the temporally symmetric form of STDP adopted here (i.e., 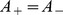 and 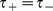). For temporally asymmetric form of STDP, we also show the similar changes of average synaptic weights in Fig. 4(b). As in the symmetric case, the feedforward structure will emerge and become stronger with the increase of the number of training trials. Differently, the incremental amount of synaptic strengths for feedforward connections and reductive amount of synaptic strengths for feedback connections are not equal (Fig. 4(b)) owing to the asymmetric modification between potentiated and depressed synaptic strengths with the same pre-post firing timing.

### Effect of Inter-stimulus Interval

**Figure 5:**
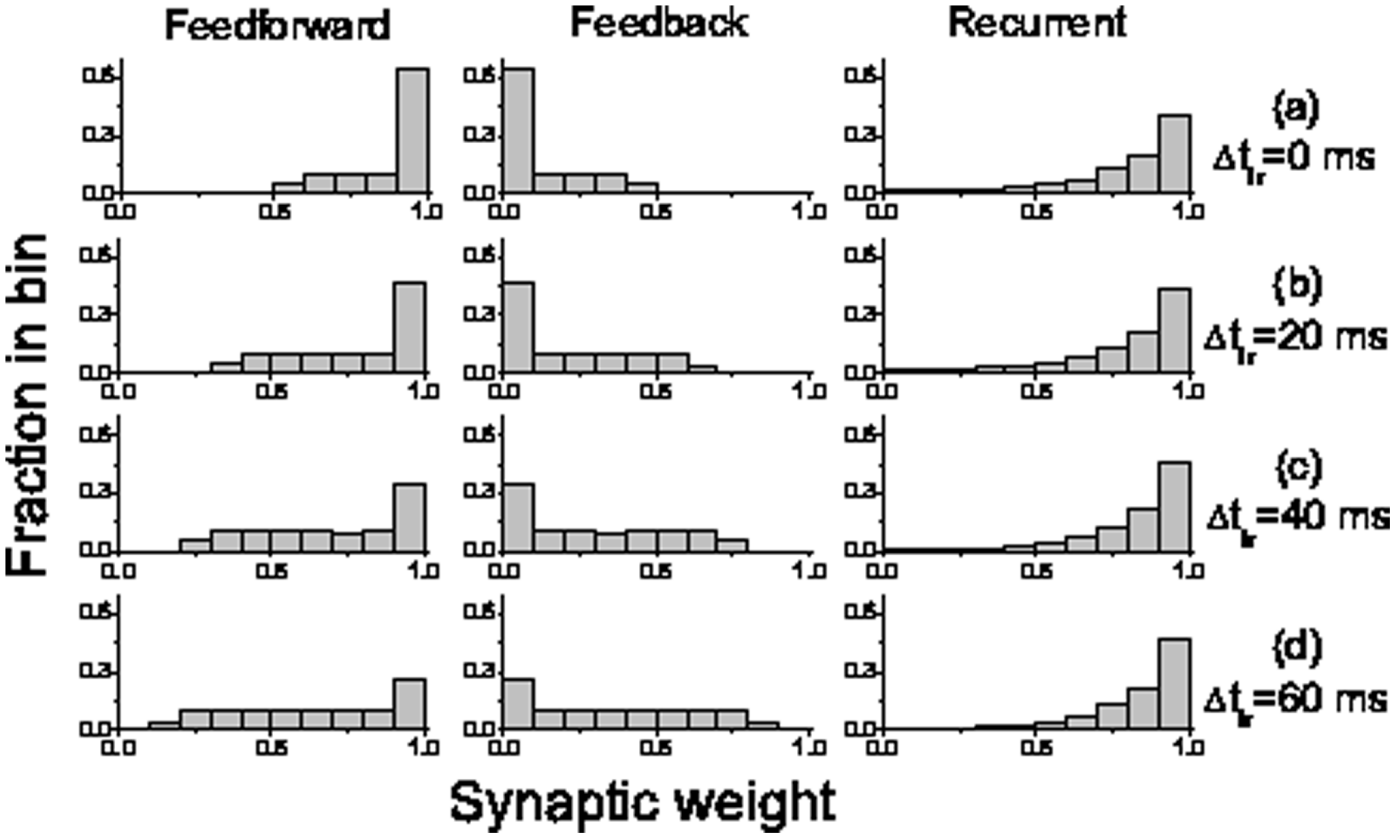
Distribution of synaptic weights after the different training stimulus intervals. Distribution of synaptic weights for different connection types: feedforward (left), feedback (middle) and recurrent (right) after the 20 training trials with different stimulus intervals 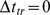 ms (a), 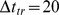 ms (b), 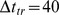 ms (c), 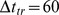 ms (d). The other parameters are the same as in Fig. 2.

We also studied the effect of inter-stimulus timing (i.e., interval 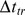) on synaptic weights for different connection types. When the interval is shortened to 0 ms (Fig. 5(a)), about 62% of feedforward synapses have been strengthened to 1 (see the left panel of Fig. 5(a)), while 62% of feedback synapses have been weakened to 0 (see the middle of Fig. 5(a)) after 20 trials, indicating a generation of strong feedforward structure (see Fig. 2(b)). With the increasing of inter-stimulus interval (Figs. 5(b)–5(d)), the resulting distributions of synaptic weights become broader, showing that the feedforward structure is weaker. For example, when the inter-stimulus interval is increased to 60 ms, there are insufficient potentiation (only 23% being approximate to 1 in the left panel of Fig. 5(d)) and insufficient depression (only 23% being approximate to 0 in the middle panel of Fig. 5(d)) after 20 trials to form feedforward structure.

The right panels of Fig. 5 show that the synaptic distribution in recurrent connections appears to be independent of inter-stimulus interval. Since the training current 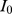 is set as a small enough value that it cannot induce spikes of neurons in other layers without receiving training, the amount of recurrent synaptic modification within layer is completely determined by duration of external current stimuli imposed to the layer (i.e., the product of 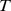 and number of training trials in Fig. 1(b)), independent of the inter-stimulus interval. This result implies that the synaptic modifications for the recurrent and feedforward/feedback connections are independent. So, the effect of recurrent connections on the formation of feedforward network can be neglected in our model.

**Figure 6:**
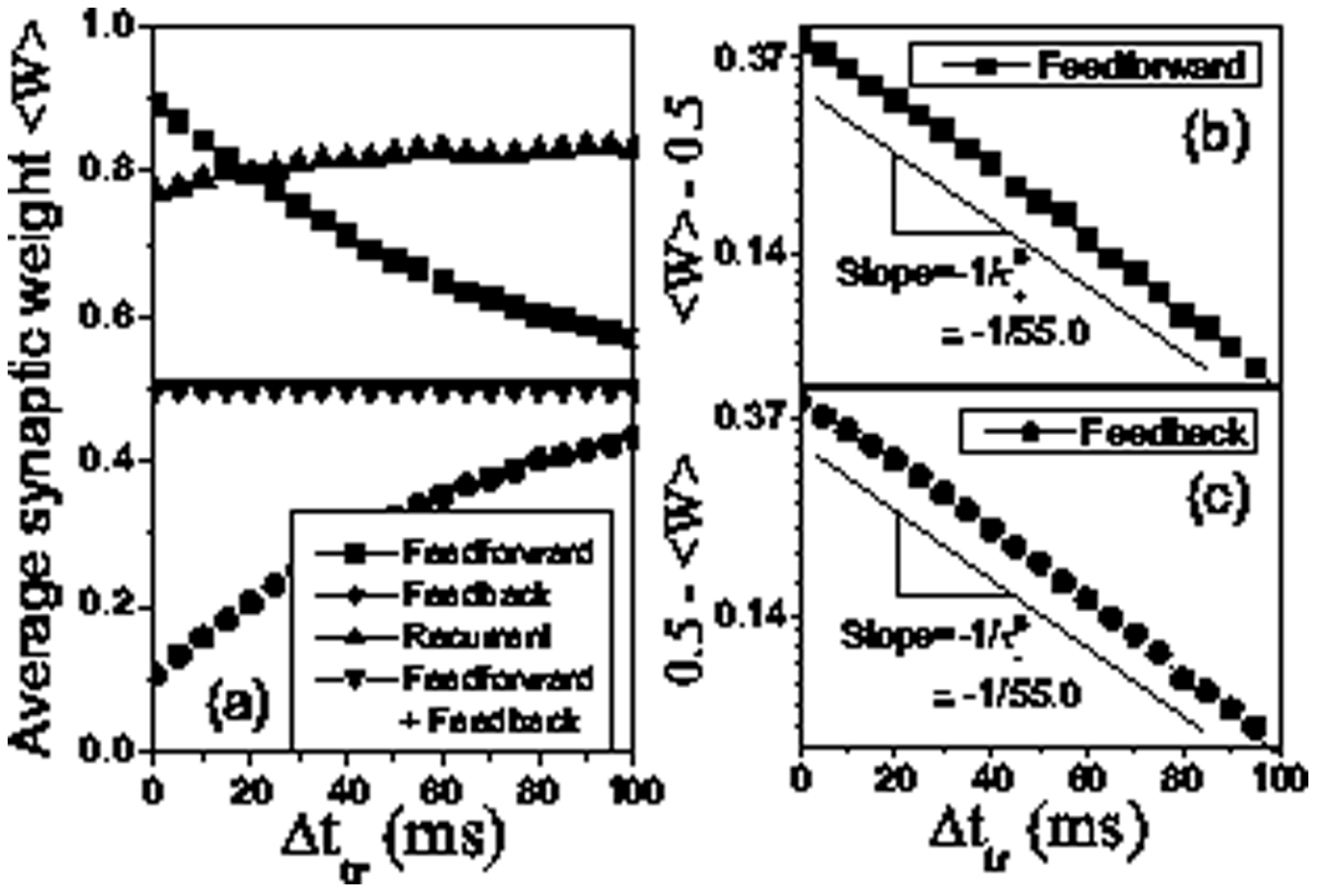
Average synaptic weights as a function of the stimulus interval for STDP with temporal symmetry. Average synaptic weights for three different connection types: feedforward (squares), feedback (circles) and recurrent (upward triangles) as a function of the stimulus interval after the 20 training trials (a). The amounts of average synaptic modification relative to its initial value 0.5, 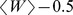 (b) and 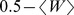 (c) for feedforward (b) and feedback (c) connections exhibit exponential falloffs as the stimulus interval 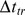 increases with exponents 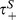 (b) and 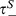 (c), respectively in linear-log scales. we also show the average synaptic weights for the connections including both feedforward and feedback types (downward triangles) in (a). The parameters are the same as in Fig. 2.

**Figure 7:**
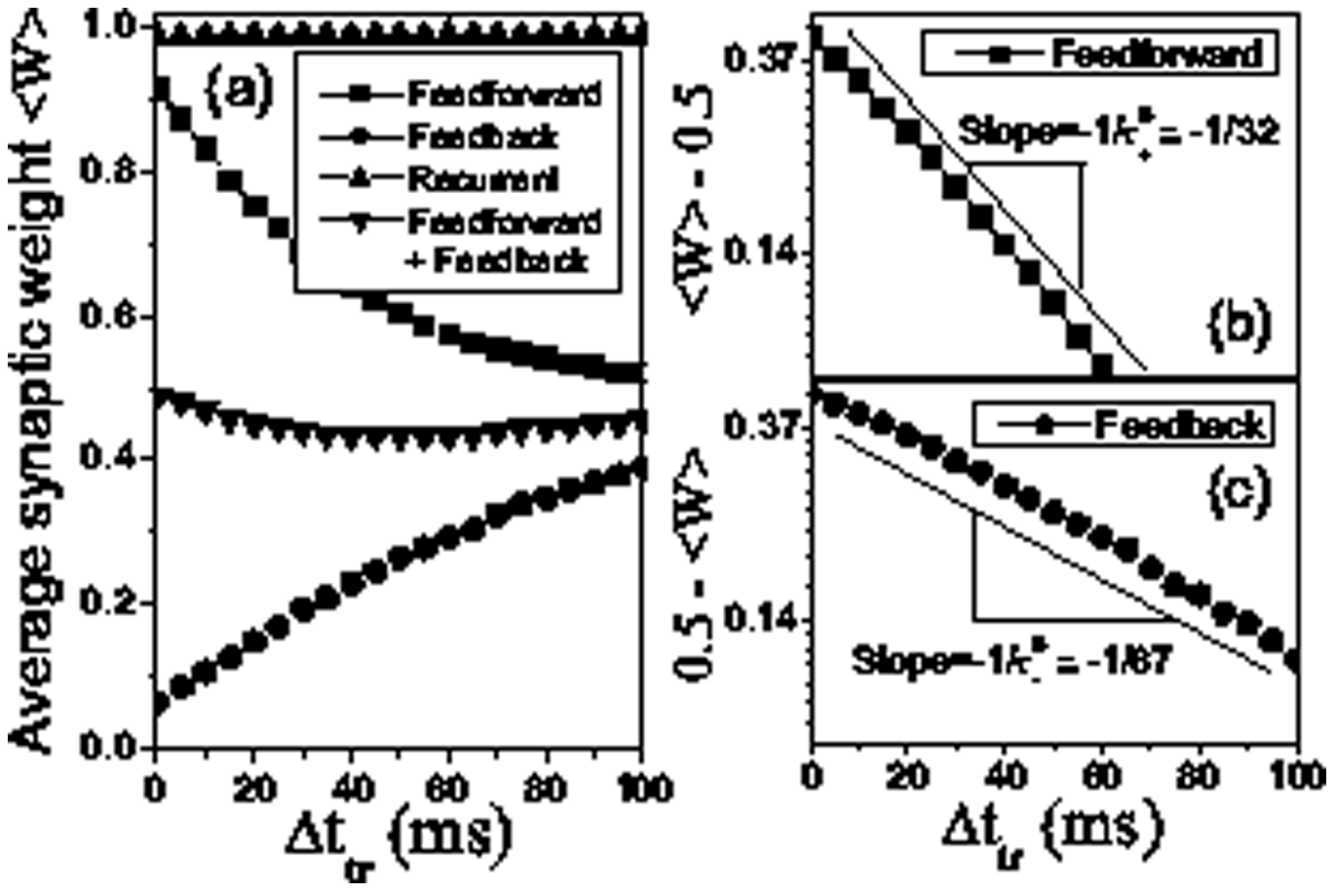
Average synaptic weights as a function of the stimulus interval for STDP with temporal asymmetry. The same as in Fig. 6, but for the temporally asymmetric form of STDP. Here, the parameters are the same as in Fig. 2, except for 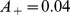, 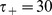 ms, 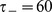 ms.

Fig. 6(a) clearly shows the changes of average synaptic weights with respect to the inter-stimulus interval. Importantly, the amount of average feedforward modification relative to its initial value 0.5 (see the value at 0 trials in Fig. 4(a)) exhibits an exponential decay with 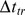, 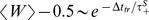 with an exponent 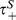 (Fig. 6(b)), and the amount of modification of average feedback connections relative to its initial value 0.5 (see the value at 0 trials in Fig. 4(a)) is also characterized as an exponential decay 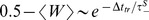 with another exponent 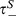 (Fig. 6(c)). For the temporally symmetric form of STDP, the two exponents 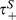 and 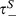 are equal (Figs. 6(b) and 6(c)) due to the equal amounts of synaptic potentiation and depression for a pre-post firing timing. The two exponents 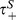 and 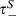 are different for the temporally asymmetric form of STDP because the symmetric modification of potentiation and depression is broken, shown in Fig. 7.

**Figure 8:**
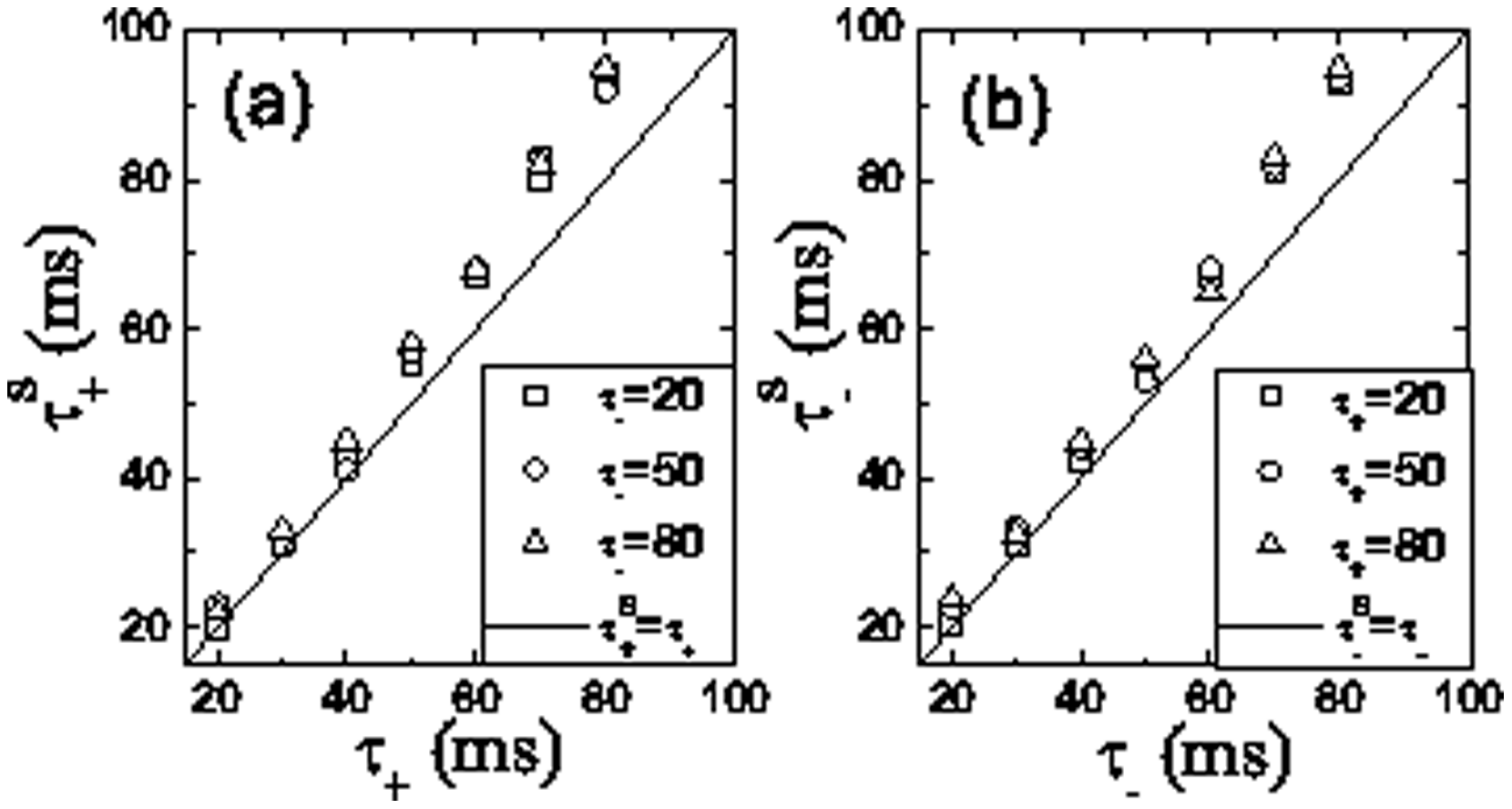
Dependance of exponents for the resulting structure on exponents of STDP after 20 training trials. (a) 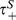 vs. 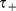 for different 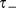 (b) 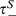 vs. 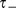 for different 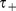. For comparison, 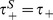 and 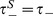 are shown as solid lines in (a) and (b), respectively. The other parameters are the same as in Fig. 2.

We numerically examined the relationships between the exponents 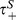 and 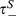 characterizing the exponential falloffs with the exponents 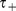 and 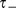 in the STDP functions (Figs. 8(a) and 8(b)). The exponents 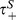 and 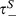 are close to 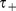 and 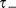, respectively. Moreover, 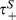 and 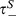 are independent of 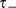 and 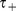, respectively. It’s further found that, these equal relationships hold for networks with different connecting parameter 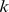 (results not shown). These findings suggest that the dependence of the structure modification on inter-stimulus interval 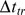 reflects the exponents of STDP. Since network structure determines dynamics of the network, a similar dependance of population dynamics on interval 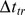 is naturally expected to reflect the underlying STDP properties in the resulting feedforward networks, which is described below.

### Propagation Property

**Figure 9:**
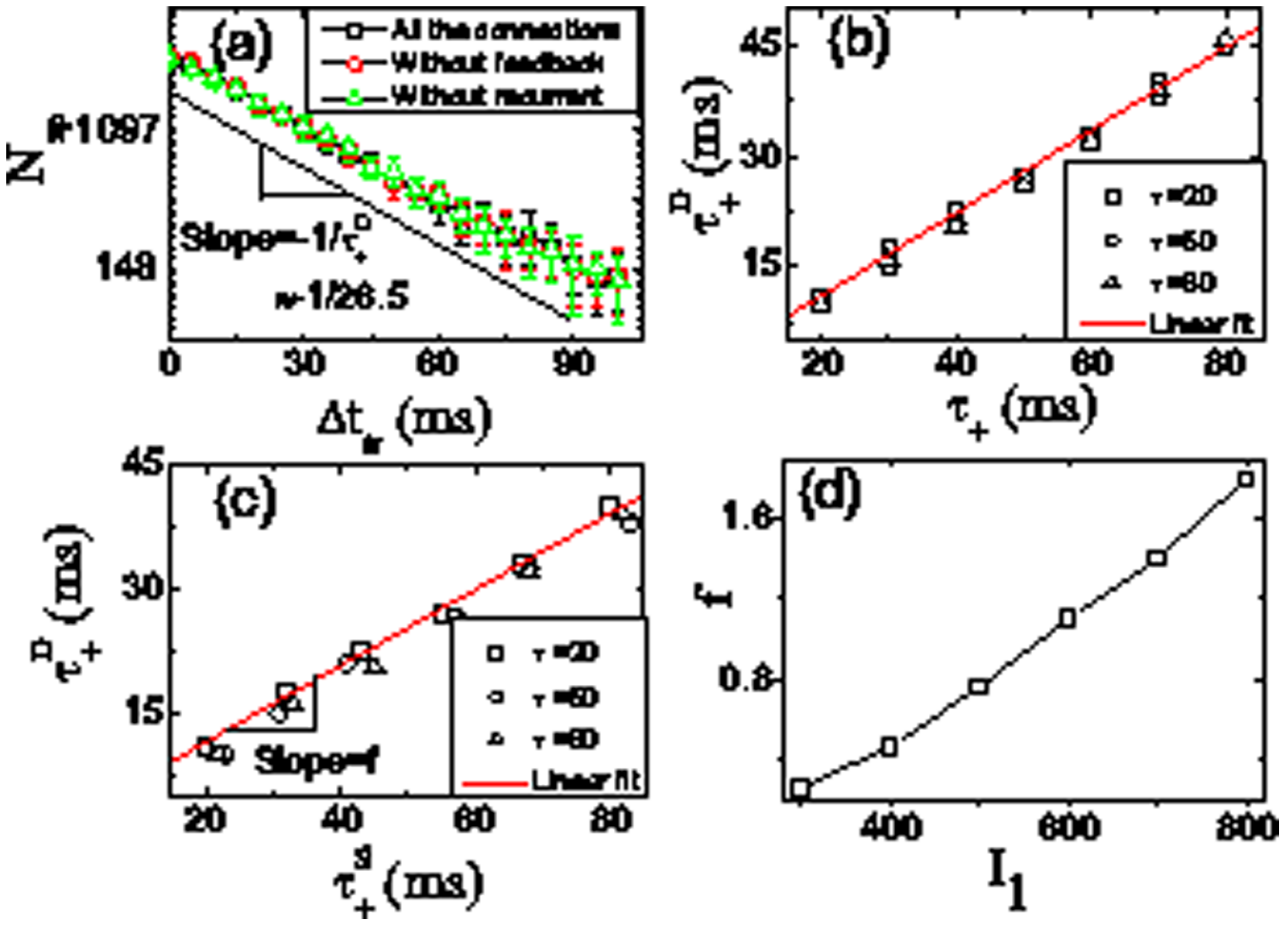
(Color online) The property of dynamical propagation in the resulting network. (a) The total number of spikes 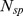 of propagated layers as a function of inter-stimulus interval 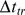 in the feedforward network obtained after 20 trials of training when keeping all the connections, indicating the exponential behavior of propagation dynamics with exponent 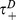 in the linear-log scales. For comparison, the 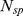 are also plotted in (a) when feedback and recurrent connections are cut (i.e., without feedback and without recurrent), respectively. (b) The exponent 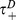 as a function of exponent 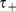 in STDP for different 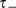. (c) The exponent 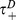 as a function of the corresponding exponent 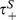 in the feedforward network structure for different 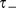. In (b) and (c), the linear fit lines of data are also given with slope 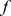. (d) The coefficient 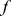 as a function of stimulation strength 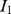. The other parameters are the same as in Fig. 2. Data of 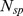 are compiled for 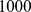 ms, and averaged over 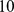 independent runs with error bar in (a).

In brain, information and cognitive processing requires signal propagation through multiple regions [39]. The feedforward network plays a crucial role in signal propagation [40]. Here we study the propagation property in the above feedforward network after training. In Fig. 9(a), we show the effect of inter-stimulus interval of training on the propagation ability. As we see in Fig. 3(c), the resulting network can support directed propagation of activity from left to right when the 3rd layer is stimulated, and the activity decays out. Here we do not consider further effect of STDP during propagation. We plot the number of spikes 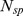 of all propagated layers (i.e., layers 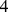 to 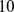) as a function of inter-stimulus interval 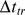. Clearly, it exhibits an exponential falloff 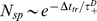 with exponent 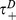, similar to the exponential falloff of STDP in Eq. (3). The exponent 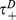 increases almost linearly with the exponent 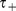 in STDP, which is almost independent of the other exponent 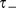 (Fig. 9(b)). This is because the spike number 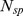 of propagated layers depends mainly on the amount of feedforward synaptic modification (not on that of feedback synaptic modification), determined by the exponent 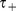 of potentiation term in Eq. (3) (Fig. 8(a)). For a comparison, we find that the scaling of the propagative activity remains the same when the feedback connections are cut (Fig. 9(a) without feedback), showing that the feedback connections are so weak that their effect on neural activity can be neglected. Additionally, the propagative activity is still almost the same without recurrent connections (Fig. 9(a)), indicating that the recurrent connections within layers also have little effect on the propagative dynamics. This is because recurrent connections are very sparse with k = 0.2 (Fig. 3(b)) although recurrent connection strengths are strong. Consequently, the resulting feedforward structure governs the propagative property of the whole network in the presence of the input current. In Fig. 9(c) we show the linear relation 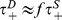 between the structure modification and dynamical propagation. The coefficient 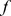 depends strongly on the stimulation strength 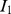. As shown in Fig. 9(d), 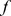 increases with 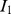. From these simulation results, we can see that a STDP-structure-dynamics relation 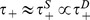 (Figs. 8(a), 9(b) and 9(c)) can emerge in the feedforward network induced by asynchronous stimuli on different spatial neuronal layers.

## Discussion

In experiments, cultured neuronal networks and developing neural systems can both generate spontaneous activity in the form of synchronized bursting events (SBEs) [41]–[43], short time events during which most of the recorded neurons fire rapidly, with several subgroups. Besides, neural activity often exhibits avalanches with some dynamical clusterings described by synchronous firing of neurons in vivo and in vitro [42], [44]–[46]. In SBEs and avalanche activity, the different dynamical clusterings behave inter-cluster asynchronous firings. So, the dynamical clusterings might strongly shape the network structure due to STDP, as the external asynchronous stimuli do in our model. This suggests an important role of STDP in generating feedforward structure and collective propagation activity on neural systems in vivo and in vitro [23], [41], [44], [45]. The role of STDP in the formation of feedforward structure driven by asynchronous stimuli might provide insight into important role of propagating feedforward (from low-level to high-level brain areas) information flow for experience-dependent map plasticity in the development of in vivo sensory pathways and cortices [4], [7], [21], [22].

The sequence learning and recall may result from activity-dependent synaptic plasticity. The conditioning-evoked sequential spiking of neighboring neurons is well suited for the induction of STDP [13], which selectively potentiate the directed connections between neurons and facilitate spike propagation in the same direction. Our study may provide an insight into formation mechanism of the sequence learning and recall. In our model, the resulting feedforward structure could denote sequence memory formed by the sequence learning. The spiking propagation in the direction of feedforward structure could reflect dynamical process of the cue-triggered recall. From Figs. 3 and 4, we can see the resulting feedforward structure will become clearer and so produce the more prominent propagation, as the number of our training trials increases. Indeed, with more repeats of stimulus sequence in experiment, the sequence learning and recall become more persistent [13]. Thus, our study suggests that STDP may modify neuronal structure and dynamics induced by external asynchronous stimuli to support the sequence learning and recall, which is consistent well with the experimental observation.

The amount of synaptic potentiation or depression due to STDP is typically measured by pairing a number of pre- and postsynaptic action potentials with a specified time interval between them [32], [33], [35], [47]. But, for a large-scaled neural system, it becomes very complex and difficult to directly test each synaptic modification due to spike timing and ordering. In our model, the resulting STDP-structure-dynamics relation (Figs. 8(a), 9(b) and 9(c)) suggests a potential method to examine STDP in large-scaled neural network. Our results demonstrate the propagative activity exponentially decays with exponent 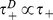, which provides an application for examining STDP by measuring neural population activity in a cultured neural network trained by external asynchronous stimuli. Cultured neural networks cultivated on multi-electrode arrays provide relatively simple and well-controlled model systems for investigating long-term activity of individual neurons at different locations [33], [41], [48]–[50]. By cultivating our two-dimensional network model and adopting our training/testing method, the strength of exponent 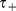 in STDP might be qualitatively examined by measuring propagation property in the resulting feedforward network. This study could shift the attention in the measurement of synaptic plasticity from the single synapse to the collective network activity. In the future, how to design other model and method for examining more parameters (e.g., 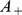, 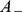 and 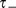) of STDP is an interesting question to pursue.

To sum up, using a simple locally linked network of integrate-and-fire neurons with STDP, we showed that it can naturally evolve into a feedforward structure when a pair of external asynchronous stimuli is used to train the network. Interestingly, the amount of synaptic modification due to asynchronous stimulus pairing with a time interval falls off exponentially as the interval increases, similar to the falloff of STDP as the interspike interval increases. Moreover, exponents (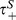 and 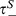) of the exponential falloff are almost the same as the exponents (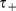 and 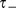) of STDP (i.e., 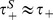 and 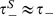). Most interestingly, propagative activity also exhibits a similar exponential falloff with exponent 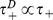 in the resulting feedforward network. Thus, by measuring the exponential falloff of the propagation activity as a function the inter-stimulus intervals in the resulting trained network, we are able to extract a direct measurement of the characteristic time scale in the network’s STDP property. Generally, STDP-structure-dynamics relation 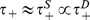 can emerge in our model. Although the formation of feedforward structure under the action of STDP has been widely studied [51], [52], the external asynchronous stimuli observed and used extensively in experiments, has not yet been investigated to induce the feedforward structure through STDP. The STDP-structure-dynamics relation is the first to be shown in the resulting feedforward network by imposing asynchronous stimuli. Our model may prove useful for understanding the relation between structure and dynamics induced by asynchronous stimuli through STDP and provide a starting point for studying the property of STDP by using external asynchronous stimuli.
