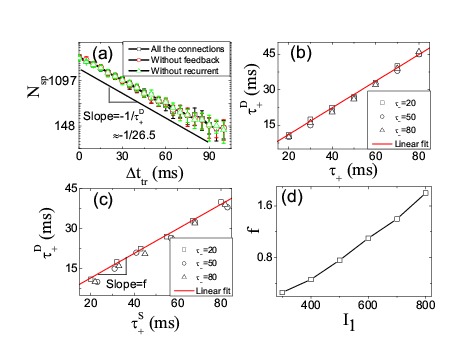# Correction: Network Evolution Induced by Asynchronous Stimuli through Spike-Timing-Dependent Plasticity

**DOI:** 10.1371/annotation/417c1eb3-1de1-4d04-8c1d-3f73ffc57f26

**Published:** 2014-01-29

**Authors:** Wu-Jie Yuan, Jian-Fang Zhou, Changsong Zhou

Multiple figures in the article contain errors. Please see corrected versions of those figures here: 

Figure 1: 

**Figure:**
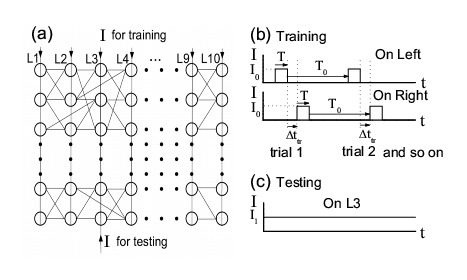


Figure 2: 

**Figure:**
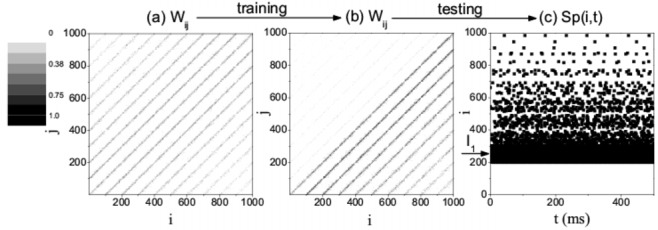


Figure 3: 

**Figure:**
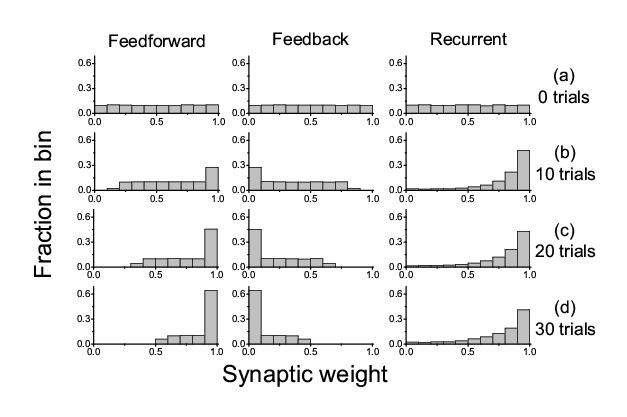


Figure 4: 

**Figure:**
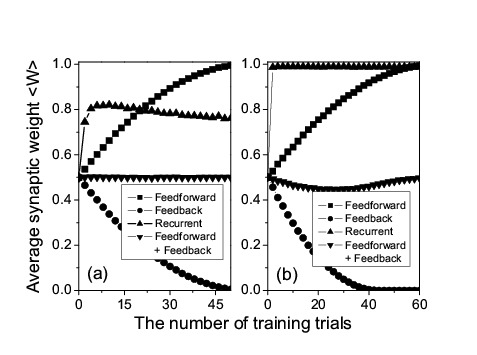


Figure 5: 

**Figure:**
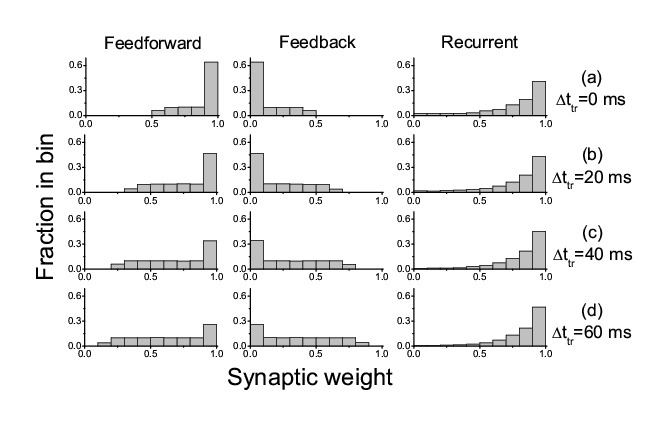


Figure 6: 

**Figure:**
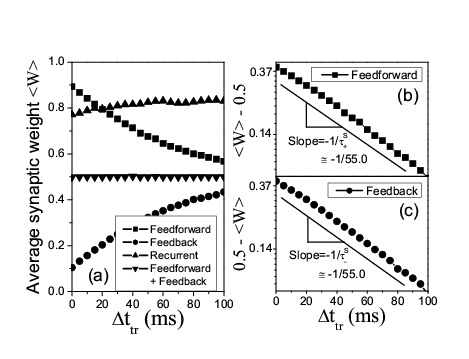


Figure 7: 

**Figure:**
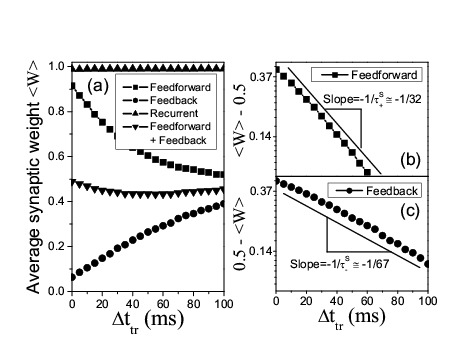


Figure 8: 

**Figure:**
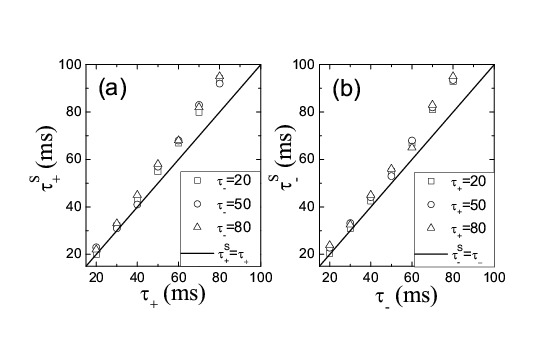


Figure 9: 

**Figure:**